# Can ischemic stroke patients with mTICI of 2b achieve similar outcomes compared to those with complete recanalization following endovascular therapy?

**DOI:** 10.3389/fneur.2024.1486586

**Published:** 2024-10-30

**Authors:** Zunbao Xu, Sahibjot Grewal, Mohammad Mofatteh, Adam A. Dmytriw, Dongqing Zhao, Baikeng Chen, Haoyang Chen, Wanyi He, Rixin Luo, Zhenzhang Li, Qiaowei Li

**Affiliations:** ^1^Neuromedical Center Ward 2, The Affiliated Panyu Central Hospital, Guangzhou Medical University, Guangzhou, China; ^2^Neurovascular Centre, Departments of Medical Imaging and Neurosurgery, St. Michael’s Hospital, University of Toronto, Toronto, ON, Canada; ^3^School of Medicine, Dentistry and Biomedical Sciences, Queen’s University Belfast, Belfast, United Kingdom; ^4^Neuroendovascular Program, Massachusetts General Hospital, Harvard Medical School, Boston, MA, United States; ^5^College of Mathematics and Systems Science, Guangdong Polytechnic Normal University, Guangzhou, China; ^6^School of Basic Medical Sciences, Guangzhou Medical University, Guangzhou, China

**Keywords:** endovascular therapy, acute ischemic stroke, mTICI, patient outcome, large vessel stroke

## Abstract

**Background and purpose:**

Endovascular therapy (EVT) has been used as a standard treatment method for patients with large vessel ischemic stroke within 24 h of the onset. The extent of recanalization after EVT can be assessed using the modified thrombolysis in cerebral infarction (mTICI) scale as an accepted angiographic grading system. In this study, we aimed to investigate whether patients with a mTICI grade of 2b achieve similar outcomes compared to those with complete recanalization (mTICI of 3) following EVT for acute ischemic stroke.

**Methods:**

A retrospective analysis was conducted on 196 consecutive patients who underwent EVT in a comprehensive stroke center. In the final study, 176 patients were included based on the inclusion criteria. The primary outcome was the 3-month modified Rankin Scale (mRS) of 0–2 considered as a favorable outcome, while excellent outcomes were defined as mRS scores of 0–1.

**Results:**

Our data showed that 59.46% of patients in the mTICI 2b group achieved a favorable outcome, comparable to 58.99% observed in the mTICI 3 group (*p* = 0.959). Additionally, 54.05% (*n* = 37) of patients with mTICI 2b achieved an excellent outcome, compared to 51.80% (*n* = 139) in the mTICI 3 group (*p* = 0.807). The case fatality rates were also comparable between the groups, with 8.11% in the mTICI 2b group and 10.79% in the mTICI 3 group (*p* = 0.632). Overall, there were no statistically significant differences between the two groups in terms of 3-month favorable outcomes, excellent outcomes, or mortality.

**Conclusion:**

Similar 3-month outcomes can be achieved for ischemic stroke patients undergoing EVT with a mTICI grade of 2b compared to those with a mTICI grade of 3. These data can help clinicians in setting realistic expectations and making informed decisions during EVT procedures.

## 1 Introduction

Endovascular therapy (EVT) has been proved as a standard treatment of choice for large vessel ischemic stroke within 24 h of the onset (1). Successful recanalization has been closely associated with favorite patient outcomes (1). The modified thrombolysis in cerebral infarction (mTICI) scale is a widely accepted angiographic grading system used to evaluate the extent of recanalization after EVT. Specifically, mTICI of 2b represents near-complete recanalization with some minor distal branch occlusions, whereas mTICI of 3 indicates complete recanalization (2).

mTICI is a widely used tool for assessing the extent of reperfusion in patients undergoing EVT. For example, assessing EVT outcomes in elderly patients with atrial fibrillation showed that patients aged 80 years and older had a lower likelihood of achieving a good functional outcome (mRS score 0–2) at 90 days and had less satisfied recanalization (mTICI, 2b-3) compared to their younger counterparts (2). In addition, a multi-institutional study of pediatric patients treated with EVT showed that successful recanalization was achieved in a significant number of cases (3). Furthermore, a multicentric Italian observational study on the endovascular treatment of acute ischemic stroke (AIS) due to tandem lesions of the anterior cerebral circulation found the procedure to be feasible, safe, and effective in improving 90-day functional outcomes (4). A recent study by Chen et al. found that early mortality occurred in 22.8% of AIS patients undergoing EVT, with recanalization status, NIHSS score 24 h post-EVT, and symptomatic intracerebral hemorrhage being significant predictors of this early mortality (1). Therefore, having a better understanding of outcomes achieved in patients with different grades of mTICI following EVT can help clinical decision-making and prognosis assessment.

While it could be considered intuitive that improved patients’ outcomes can be associated with a higher recanalization rate, there is lack of consensus on the most appropriate scale on different grades of TICI. A further challenge arises from the fact that the definition of different mTICI grades lacks distinct parameters to enable replicating results across various stroke centers. Therefore, the distinction between mTICI 2b and 3 as well as their implications for patient outcomes remains a topic of interest in stroke research. The aim of this study was to investigate the differences in outcomes between patients with mTICI grades of 2b and 3 after EVT, specifically, addressing whether patients with a mTICI grade of 2b could achieve outcomes similar to those with complete recanalization following EVT.

## 2 Methods

### 2.1 Patient population

A retrospective analysis of prospectively collected data was conducted on consecutive patients who underwent EVT in a comprehensive stroke center in China between January 2019 to April 2022. The hospital provide service for more than 2.8 million people. The period observation ranged from. Inclusion criteria were as following: (1) age ≥ 18 years old; (2) mTICI of 2b-3, (3) within 24 h from last know well time (LKW), (4) had premorbid modified Rankin Scale (mRS). Exclusion criteria were as follows: (1) patients without follow-up, (2) over 24 h from LKW, (3) with mTICI <2b.

### 2.2 Data collection

We collected the baseline information of patients, risk factors, premorbid mRS, time metrics of thrombolysis and EVT, pre-EVT National Institute of Health stroke scale (NIHSS), occlusion vascular sites, symptomatic intracranial hemorrhage (sICH), pre-EVT Alberta stroke program early CT score (ASPECTS). The post EVT recanalization was evaluated by mTICI. The degree of recanalization was assessed using the Modified Thrombolysis in Cerebral Infarction (mTICI) scale, with scores assigned by a trained radiologist. The mTICI scale grades were determined based on the degree of reperfusion observed in the post-procedural angiograms, with a score of 2b indicating near-complete recanalization and a score of 3 indicating complete recanalization. Clinical outcomes were retrospectively evaluated using the modified Rankin Scale (mRS) at 3 months post-procedure. Favorable outcomes were defined as mRS scores of 0–2, indicating functional independence, while excellent outcomes were defined as mRS scores of 0–1. The mRS scores were collected during routine follow-up evaluations and were retrospectively reviewed in this study (5, 6).

### 2.3 Statistical analysis

The non-parametric Mann–Whitney U test was performed using IBM SPSS 26 version (IBM-Armonk, NY) to analyze non-normally distributed continuous data, reported as medians along with the interquartile range (IQR). categorical variables were described as frequencies and percentages. Results were considered statistically significant if the *p*-value was less than 0.05.

### 2.4 Ethics approval and consent to participate

The study protocol was approved by the hospital institutional review board. Informed consents were waived due to the retrospective nature of the study in compliance with national laws and regulations. All procedures performed in the studies involving human participants were in accordance with the ethical standards of the institutional and/or national research committee and with the 1964 Declaration of Helsinki and its later amendments or comparable ethical standards.

## 3 Results

### 3.1 Participant selection

In total, 196 patients who underwent EVT were initially evaluated for eligibility. Twenty patients were excluded for the following reasons: three patients with LKW time over 24 h, 11 patients were mTICI <2b, and six patients were lost to follow-up. 176 patients were eligible and included in the final study.

### 3.2 Study characteristics

Our analysis of baseline characteristics between patients with mTICI scores of 2b (*n* = 37) and 3 (*n* = 139) revealed notable similarities and differences. In the mTICI 2b group, 43.24% were females, with a median age of 65 years (IQR 55–73.5) and a hypertension prevalence was 54.05%. In contrast, the mTICI 3 group had a slightly lower female percentage of 38.85% with a comparable median age of 65 years (IQR 58–75).

The frequency of hypertension, diabetes, coronary artery disease, atrial fibrillation, stroke history, smoking, and hyperlipemia showed no significant statistical differences between the two groups. The median pre-EVT NIHSS scores and pre-EVT ASPECTS were also similar between the groups, indicating comparable severity and the extent of stroke at the baseline. The statistical analysis demonstrated no significant differences in these baseline characteristics, highlighting the comparability of the two patient groups at the onset of their treatment (Table 1).

**Table 1:** Comparison of baseline characteristics mTICI of 2b and 3.

	mTICI of 2b (*n* = 37)	mTICI of 3 (*n* = 139)	*p*
Female, *n* %	16 (43.24%)	54 (38.85%)	0.627
Age year (IQR)	65.000 (55.0,73.5)	65.000 (58.0,75.0)	0.371
Hypertension, *n*, %	20 (54.05%)	88 (63.31%)	0.304
Diabetes, *n*, %	5 (13.51%)	31 (22.30%)	0.239
Coronary artery disease, *n*, %	3 (8.11%)	14 (10.07%)	0.719
Atrial fibrillation, *n*, %	17 (45.95%)	61 (43.88%)	0.823
Stroke, *n*, %	6 (16.22%)	26 (18.71%)	0.727
Smoker, *n*, %	8 (21.62%)	33 (23.74%)	0.786
Hyperlipemia	14 (37.84%)	52 (37.41%)	0.962
Pre-EVT NIHSS (IQR)	12.000 (8.0,19.0)	13.000 (9.0,18.0)	0.613
Pre-EVT ASPECTS	10.000 (9.0,10.0)	10.000 (9.0,10.0)	0.646
ICA, *n*, %	4 (10.81%)	30 (21.58%)	0.187
MCA-M1, *n*, %	18 (48.65%)	53 (38.13%)	
MCA-M2, *n*, %	7 (18.92%)	12 (8.63%)	
Tandem (ICA + M1), *n*, %	4 (10.81%)	13 (9.35%)	
Others	3 (8.11%)	21 (15.11%)	
Bridging thrombolysis, *n*, %	22 (59.46%)	64 (46.04%)	0.147
DNT	46.000 (38.0, 59.0)	45.000 (38.0, 59.0)	0.848
ONT	148.000 (124.0, 186.0)	140.000 (101.3, 173.3)	0.372
DPT (IQR), min	104.000 (81.0, 121.0)	100.000 (80.5, 122.0)	0.567
DRT (IQR), min	149.000 (129.5, 203.0)	140.000 (114.0, 189.0)	0.134
LKWPT (IQR), min	276.000 (200.5, 360.5)	264.000 (172.0, 466.0)	0.839
sICH, *n*, %	1 (2.70%)	6 (4.32%)	0.655

ASPECTS, Alberta stroke program early CT score; DNT, door-to-needle time; DPT, door-to-puncture time; DRT, door-to-recanalization time; EVT, Endovascular therapy; ICA, internal carotid artery; IQR, interquartile range; LKWPT, last-known well-to-puncture time; MCA, middle cerebral artery; NIHSS, National Institute of Health stroke scale; ONT, onset-to-needle time; sICH, symptomatic intracranial hemorrhage.

### 3.3 3-month outcomes

**Figure 1:**
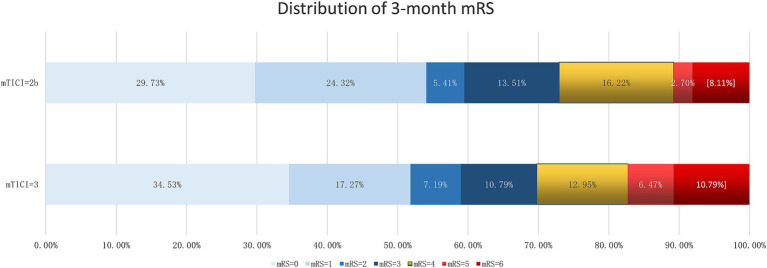
Distribution of 3-month mRS between mTICI = 2b and mTICI = 3.

Comparing the 3-month outcomes of patients with mTICI scores of 2b (*n* = 37) and 3 (*n* = 139), our findings showed no significant differences in excellent outcome, mortality, and favorable outcome rates between these two groups. Specifically, 54.05% of patients in the mTICI 2b group achieved an excellent outcome, compared to 51.80% in the mTICI 3 group (*p* = 0.807). Case fatality rates were slightly lower in the mTICI 3 group (10.79%) compared to the mTICI 2b group (8.11%), but this difference was not statistically significant (*p* = 0.632). Favorable outcomes were observed in 59.46% of the mTICI 2b group and 58.99% of the mTICI 3 group, with no statistically significant differences (*p* = 0.959; Table 2, Figure 1).

**Table 2:** Comparison of 3-month outcome of 2b and 3.

	mTICI of 2b (*n* = 37)	mTICI of 3 (*n* = 139)	*p*
Excellent outcome, n, %	20 (54.05%)	72 (51.80%)	0.807
Mortality, n, %	3 (8.11%)	15 (10.79%)	0.632
Favorable outcome, n, %	22 (59.46%)	82 (58.99%)	0.959

mTICI, modified thrombolysis in cerebral infarction.

## 4 Discussion

The primary objective of our study was to investigate whether patients with a mTICI grade of 2b achieve similar outcomes as those with complete recanalization (mTICI 3) following EVT. Our findings suggest that there is no significant difference in 3-month excellent outcome, favorable outcome, or mortality between these two groups.

EVT has emerged as a standard treatment for large vessel ischemic stroke within the first 24 h after onset (1). The degree of recanalization, as measured by the mTICI scale, has been closely associated with patient outcomes in various studies (7). Our inclusion and exclusion criteria ensured a focused analysis on patients who underwent EVT within the recommended time frame and achieved near-complete to complete recanalization.

While both mTICI 2b and mTICI 3 have traditionally been considered as indicators of successful reperfusion, recent studies have investigated the distinctions between these grades. Another study emphasized the significance of achieving reperfusion rapidly on the first pass, suggesting that grades of TICI 2c-3 might be more desirable endpoints in EVT due to their association with improved clinical outcomes compared to TICI 2b (8). Another investigation underscored the benefits of redefining successful reperfusion as TICI 2c/3, indicating that patients with a TICI 3 exhibited evidence of faster recovery and superior outcomes compared to those with the traditional TICI 2b (9). This distinction was further supported by a network meta-analysis, which found that TICI 2C and 3 were associated more significantly with favorable 90-day clinical outcomes than TICI 2B. Moreover, there was no discernible difference in outcomes between TICI 2C and 3 (10).

The findings in our study challenges the reported results of the aforementioned studies, which held view that a mTICI grade of 3 is superior to a grade of 2b in terms of clinical outcomes following EVT for AIS. We observed that patients with a mTICI grade of 2b achieved outcomes that were not significantly different from those with a grade of 3. This challenges the prevailing notion that only a grade of 3 constitutes a successful EVT outcome, highlighting the efficacy of achieving a 2b grade in clinical practice. Results from our study, in combination with other factors, can be used to establish models to predict patient outcome and prognosis (1, 11–14).

This observation is particularly noteworthy considering the clinical implications of EVT, suggesting that while striving for complete recanalization remains a valid target, achieving a grade of 2b should also be recognized as a successful outcome of EVT. Therefore, these findings could potentially broaden the scope of successful EVT, offering reassurance to patients and clinicians, especially in cases where complete recanalization might not be feasible.

While our study provides valuable insights into the outcomes of EVT in AIS, it is important to consider its limitations. As a retrospective analysis, the data are inherently subject to potential biases commonly associated with retrospective data collection methods. In addition, the study was conducted in a single comprehensive stroke center in China, which may limit the generalizability of our findings to other populations or healthcare settings. The relatively small sample size of the TICI 2B group and TICI 3 group may also limit the statistical power to detect smaller differences between these groups, potentially underestimating distinctions in clinical outcomes. Furthermore, many factors can influence post-EVT outcomes, some which we accounted for, such as baseline patient health, risk factors, premorbid mRS, time metrics of thrombolysis and EVT, pre-EVT NIHSS scores, occlusion vascular sites, and the occurrence of complications like symptomatic intracranial hemorrhage (sICH). Although our study accounted for these variables, the potential for residual confounding factors remains. This recognition underscores the need for caution in interpreting our findings and highlights the complexity of determining the success of reperfusion therapy in AIS patients.

The findings of our study can pave the way for future investigations in stroke management and EVT. Future investigations should focus on multi-center, prospective studies to validate and extend our findings across diverse demographics and clinical settings. These studies could shed lights into understanding the interplay between various mTICI grades and patient outcomes, particularly examining the mechanisms that lead to favorable results in patients with a mTICI grade of 2b. Additionally, long-term follow-up studies are crucial to evaluate the sustained impacts of different mTICI grades on patient recovery and quality of life.

## 5 Conclusion

This study’s findings highlight that achieving a mTICI grade of 2b is comparably effective as reaching a mTICI grade of 3 in terms of 3-month outcomes for AIS patients undergoing EVT. Specifically, the similar rates of excellent outcomes, mortality, and favorable outcomes between the two groups underscore the therapeutic value of EVT, even when complete recanalization (mTICI of 3) is not achieved. These results offer reassurance that mTICI 2b outcomes lead to positive patient prognoses and offers insight to clinicians to make informed decisions during EVT procedures.

## Data Availability

The raw data supporting the conclusions of this article will be made available by the authors, without undue reservation.

## References

[1] Yoshimura S Sakai N Yamagami H Uchida K Beppu M Toyoda K. Endovascular therapy for acute stroke with a large ischemic region. N Engl J Med. (2022) 386:1303–13. doi: 10.1056/NEJMoa2118191 35138767

[2] Jiao J Liu S Cui C Cao Y Jia Z Liu H. Endovascular thrombectomy for acute ischemic stroke in elderly patients with atrial fibrillation. BMC Neurol. (2022) 22:100. doi: 10.1186/s12883-022-02631-3, PMID: 35300621 PMC8928604

[3] Ravindra VM Alexander M Taussky P Bollo RJ Hassan AE Scoville JP. Endovascular Thrombectomy for pediatric acute ischemic stroke: a multi-institutional experience of technical and clinical outcomes. Neurosurgery. (2020) 88:46–54. doi: 10.1093/neuros/nyaa312, PMID: 32761237 PMC8660626

[4] Bracco S Zanoni M Casseri T Castellano D Cioni S Vallone IM. Endovascular treatment of acute ischemic stroke due to tandem lesions of the anterior cerebral circulation: a multicentric Italian observational study. Radiol Med. (2021) 126:804–17. doi: 10.1007/s11547-020-01331-7, PMID: 33502665 PMC8154792

[5] ElHabr AK Katz JM Wang J Bastani M Martinez G Gribko M. Predicting 90-day modified Rankin scale score with discharge information in acute ischaemic stroke patients following treatment. BMJ Neurol Open. (2021) 3:e000177. doi: 10.1136/bmjno-2021-000177, PMID: 34250487 PMC8231000

[6] Banks JL Marotta CA. Outcomes validity and reliability of the modified Rankin scale: implications for stroke clinical trials. Stroke. (2007) 38:1091–6. doi: 10.1161/01.STR.0000258355.23810.c6 17272767

[7] Tung EL McTaggart RA Baird GL Yaghi S Hemendinger M Dibiasio EL. Rethinking thrombolysis in cerebral infarction 2b. Stroke. (2017) 48:2488–93. doi: 10.1161/STROKEAHA.117.017182, PMID: 28775136

[8] Yoo AJ Soomro J Andersson T Saver JL Ribo M Bozorgchami H. Benchmarking the extent and speed of reperfusion: first pass TICI 2c-3 is a preferred endovascular reperfusion endpoint. Front Neurol. (2021) 12:669934. doi: 10.3389/fneur.2021.669934, PMID: 34046008 PMC8144635

[9] Almekhlafi MA Mishra S Desai JA Nambiar V Volny O Goel A. Not all “successful” angiographic reperfusion patients are an equal validation of a modified TICI scoring system. Interv Neuroradiol. (2014) 20:21–7. doi: 10.15274/INR-2014-10004, PMID: 24556296 PMC3971136

[10] Jang KM Nam TK Ko MJ Choi HH Kwon JT Park SW. Thrombolysis in cerebral infarction grade 2C or 3 represents a better outcome than 2B for endovascular Thrombectomy in acute ischemic stroke: a network Meta-analysis. World Neurosurg. (2020) 136:e419–39. doi: 10.1016/j.wneu.2020.01.020 31931242

[11] Lai Y Diana F Mofatteh M Nguyen TN Jou E Zhou S. Predictors of failure of early neurological improvement in early time window following endovascular thrombectomy: a multi-center study. Front Neurol. (2023) 14:1227825. doi: 10.3389/fneur.2023.1227825, PMID: 37780716 PMC10538528

[12] Lai Y Jou E Mofatteh M Nguyen TN Ho JSY Diana F. 7-day National Institutes of Health stroke scale as a surrogate marker predicting ischemic stroke patients’ outcome following endovascular therapy. Transl Neurosci. (2023) 14:20220307. doi: 10.1515/tnsci-2022-0307, PMID: 37873059 PMC10590605

[13] Ždraljević M Pekmezović T Stanarčević P Vukašinović I Berisavac I Ercegovac M. Atrial fibrillation is associated with poor long-term outcome after mechanical thrombectomy for anterior large vessel occlusion stroke. J Stroke Cerebrovasc Dis. (2022) 31:106755. doi: 10.1016/j.jstrokecerebrovasdis.2022.106755, PMID: 36191566

[14] Diprose WK Liem B Wang MTM Sutcliffe JA Brew S Caldwell JR. Impact of body temperature before and after endovascular Thrombectomy for large vessel occlusion stroke. Stroke. (2020) 51:1218–25. doi: 10.1161/STROKEAHA.119.028160, PMID: 32102631

